# To Eat or Not To Eat? The Diet of the Endangered Iberian Wolf (*Canis lupus signatus*) in a Human-Dominated Landscape in Central Portugal

**DOI:** 10.1371/journal.pone.0129379

**Published:** 2015-06-01

**Authors:** Rita Tinoco Torres, Nicole Silva, Gonçalo Brotas, Carlos Fonseca

**Affiliations:** 1 Department of Biology & CESAM, University of Aveiro, Campus de Santiago, Aveiro, Portugal; 2 Associação de Conservação do Habitat do Lobo Ibérico, Rua 25 de Abril, Esposende, Portugal; University of Queensland, AUSTRALIA

## Abstract

Livestock predation by large carnivores and their persecution by local communities are major conservation concerns. In order to prevent speculations and reduce conflicts, it is crucial to get detailed and accurate data on predators’ dietary ecology, which is particularly important in human dominated landscapes where livestock densities are high. This is the case of the endangered Iberian wolf in Portugal, an endemic subspecies of the Iberian Peninsula, which has seen its population distribution and abundance decline throughout the 20th century. Accordingly, the diet of the Iberian wolf was analyzed, using scat analysis, in a humanized landscape in central Portugal. From 2011 to 2014, a total of 295 wolf scats were collected from transects distributed throughout the study area, prospected on a monthly basis. Scat analysis indicated a high dependence of Iberian wolf on livestock. Domestic goat predominated the diet (62% of the scats), followed by cow (20%) and sheep (13%); the only wild ungulate present in the scat analysis was the wild boar (4% of the scats). Our results show that even though livestock constitute most part of wolves diet, different livestock species may represent different predation opportunities. We conclude that the high levels of livestock consumption may be a result of low diversity and density of wild ungulates that settles livestock as the only abundant prey for wolves. Our findings help on the understanding of the Iberian wolf feeding ecology and have implications for conflict management strategies. Finally, management implications are discussed and solutions are recommended.

## Introduction

Top predators are often considered flagship species and conservation tools for the whole biological diversity of its supporting ecosystem [1]. The grey wolf (*Canis lupus*) is an important top predator and can play a key role in maintaining biodiversity in ecosystems [2]. Even though by the end of the 19^th^ century this species was exterminated from most central and northern European countries, it now has one of the largest distribution ranges among large carnivore species [3]. Contrarily, the Iberian wolf (*Canis lupus signatus*), an endemic subspecies of the Iberian Peninsula, has seen its population distribution and abundance decline throughout the 20^th^ century, mostly in Portugal, where its numbers have plummeted and its range has massively contracted [4]. This subspecies is protected in Portugal by law since 1988 being listed as “Endangered” in the Portuguese Red Data Book [5]. Primary threats to wolf survival include habitat degradation and fragmentation, scarcity of wild prey with consequent livestock predation, and illegal persecution primarily in retaliation for predation on livestock [4].

The feeding ecology of the wolf has been extensively investigated across its European range: in central and north-eastern Europe, wolves feed mainly on wild ungulates such as red deer (*Cervus elaphus*), roe deer (*Capreolus capreolus*) and wild boar (*Sus scrofa*) [6], [7], [8], [9], [10], [11], [12]. Contrastingly, in south Europe, which is characterized by areas with high human density and intense livestock production, wolves seem to depend largely on anthropogenic food sources such as livestock and garbage [13], [14], [15], [16], [17], [18]. Many studies have shown that large carnivores can persist in human-dominated areas by relying completely or partially on anthropogenic food resources [19], but further complications may arise from such increased interaction. Livestock predation is one of the main rooted conflicts between humans and top predators particularly in areas with low densities of wild prey where predators depend almost solely on livestock [20], [21]. Predation on livestock has been the trigger for conflict with rural communities in various parts of the world and has a negative impact on the economy of rural communities that coexist with it [22]. This has shown to be very relevant in Portugal, since livestock is the main economic activity of the rural mountain communities [6]. Each year there are reports on livestock losses from wolf depredation in areas inhabited by the Iberian wolf in Portugal. Currently, the annual amount of compensation is around € 1,000,000 corresponding to about 2,400 attacks attributed to the wolf (R. Rodrigues, personal communication). Wolf predation on livestock is regarded as the main struggle between Iberian wolf and the local populations and it boosts the already rooted conflict with humans, which is ultimately translated high levels of wolves mortality, most of them man-related (*e*.*g*. shooting, poisoning or trapping) [4]. In order to prevent speculations and reduce conflicts between wolves and local communities, it is pivotal to get detailed and accurate data on predators diet. A clear understanding of this endangered carnivore diet is essential for the design of tangible conservation strategies, particularly in areas where the conflict exists. Diet studies are particularly important when an animal is elusive, with low densities and difficult to observe and follow, such as the Iberian wolf. Nevertheless, to date, despite numerous studies on wolf diet elsewhere in Europe, few works have dealt with the diet of the Iberian wolf, particularly in Portugal (but see [14], [17]). To improve our understating of resource use by the Iberian wolf in a human-dominated landscape, we analyzed the diet of Iberian wolf in central Portugal from 2011 to 2014. This study aims to answer the following questions about the Iberian wolf population: i) does livestock constitute the main part of the Iberian wolf diet? and ii) does the Iberian wolf show diet selection towards any livestock species? Since adequate knowledge of dietary habits is essential to understand the ecology of this species and for the development of an appropriate scientific based management plan, we also discuss the general implications of our findings. As the densities of livestock are high and wild ungulates are not abundant in our study area, we hypothesize that wolves on the south of the Douro river will mainly feed on livestock. Our results provide important knowledge for the conservation and management of this endangered species.

## Material and Methods

### Ethics Statement

Our research did not involve capture, handling or killing of animals, therefore did not require approval of animal care and use procedures. Permissions for field studies were given by Nature and Forestry Conservation Institute.

### Study area

**Fig 1:**
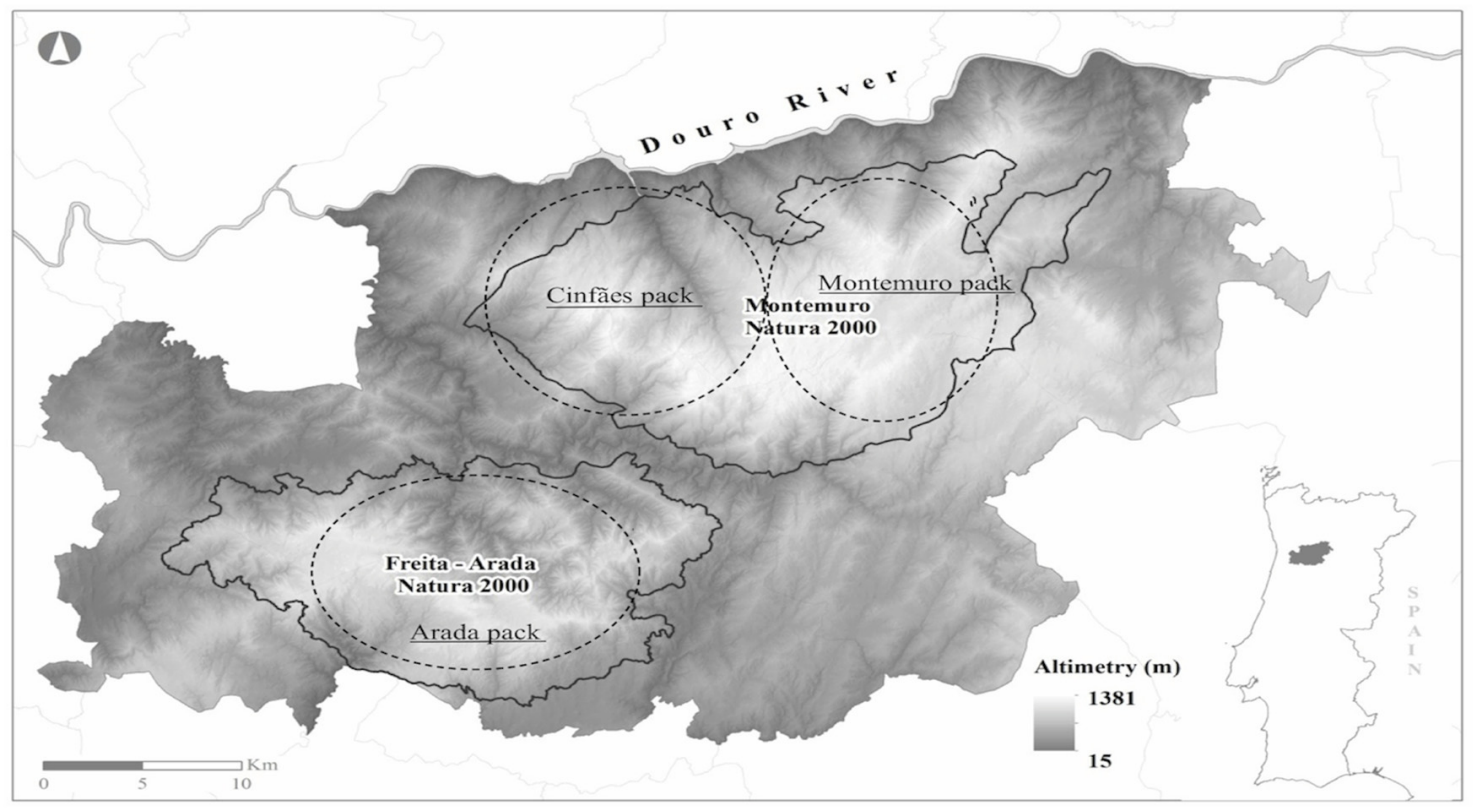
Location of the study area in continental Portugal, highlighting the distribution of the three Iberian wolf packs.

The study was conducted in central-west Portugal in two Natura 2000 network sites, with an area of 750 km^2^ (Fig 1). It is a mountainous region with altitudes ranging from 800 to 1,381m and steep slopes. The climate is mainly Mediterranean, with strong oceanic influence. The study area is composed mainly by forests (46%), scrubland (26%), agricultural land (20%) and urban area (8%). The vegetation is diverse and is mainly constituted by different types of scrublands (e.g. *Cytisus scoparius*, *Cytisus grandiflorus*, *Ulex* spp., *Genista triacanthos*, *Erica* spp., and *Pterospartum tridentatum*). The trees present in the area are the English oak *Quercus robur*, the Pyrenean oak *Quercus pyrenaica*, the sweet chestnut *Castanea sativa*, the Maritime pine *Pinus pinaster*, in pure stands or mixed with the eucalyptus *Eucalyptus globulus*. Scattered pastures and agricultural fields can still be found along the study area, which is crossed by several rivers and streams. The riparian vegetation is mainly constituted by ash *Fraxinus angustifolia* and birch *Betula alba*. The wild boar is the only wild ungulate that inhabits the study area, while the domestic ungulates present are goats (*Capra hircus*), sheep (*Ovis aries*), horses (*Equus ferus caballus*) and cattle. Other mammals present are the red fox (*Vulpes vulpes*). In the study area, man still subsists on agriculture and pastoralism. Extensive livestock of native cattle and small ruminants predominates, with extensive use of vacant land. Livestock grazes under the traditional grazing system. Sheep, goat and cow flocks range around all over the mountains in unfenced areas. Sheep flocks generally graze together with the presence of a shepherd and/or guarding dogs. Goats tend to disperse across the mountains, sometimes with the presence of a shepherd and/or guarding dogs. Cows graze alone as shepherds have the practice of leaving these animals to range freely all year round. All the livestock species in our study area spend the night in barns. Traditionally, there were transhumant herds in our study area, but transhumance disappeared therefore livestock is available all year round. In the region that comprehends the Arada pack, livestock is composed by 60% of sheep, 27% of goats and 13% of cows; in the Montemuro pack, livestock is composed by 54% of sheep, 31% of goats and 15% of cows and in the Cinfães pack, livestock is composed by 50% of sheep, 24% of goat and 26% of cows. These numbers are based on the national census of agriculture [23]. The human population is dispersed through the valleys, in small villages with a population density of about 43 inhabitants/km^2^.

### Scat collection

In Portugal the Iberian wolf occurs in an area of about 20,000 km^2^. The genetic studies have demonstrated the existence of two seemingly isolated subpopulations, separated by the river Douro [24]. The packs on the north of the river Douro are considered to be stable with an apparent local expanding trend, particularly in areas close to the borders with Spain. On the contrary, the packs located on the south side of this river (only 6 confirmed packs) are isolated from the remaining populations (*e*.*g*. north of Douro river and the Spanish population), showing a high level of fragmentation and low genetic diversity [24]. The subpopulation on the south of the Douro river is distributed in an area of about 3.800 km^2^ with an average density of 0.5 to 1.3 per 100km^2^ [4]. Our study area includes the range occupied by three of these six confirmed southern wolf packs: Arada pack, Montemuro pack and Cinfães pack (Fig 1). Wolf scats were systematically collected between October 2011 and April 2014 on a monthly basis in predefined transects. Scats were collected along existing trails, by foot or using a vehicle (< 10 km/h), following paths, dirt roads, forest trails, firebreaks and crossroads, by experienced and field-trained personnel. As wolves occur at low densities, with the possibility of many transects with no signs or tracks, a large number of transects were randomly distributed along the study area. A total of 47 transects were distributed throughout the study area, and were prospected each month, which corresponds to 130.4 km (smallest transect: 0.6 km; largest transect: 7.4 km). We also collected scats opportunistically while travelling to and between transects. Wolf scats were stored in plastic bags and always properly labeled for later processing. In addition, a field form was recorded and scat locations were registered using a GPS (*Global Positioning System*).

Scats analysis constitute the most readily available and easily collected source of diet information and has been previously used to study carnivore’s diet worldwide, particularly wolf [25]. Such technique is based on the identification of undigested remains (*e*.*g*. bones, hairs, etc) in scats [25]. Some authors have raised some methodological limitations of this technique as they claim that large prey bones and teeth are generally fragmented in the predator scats and therefore difficult to identify [26] and the hairs from the same prey animal may vary in structure according to their location and related species may have hair with similar characteristics [26]. In order to address this problem, some studies have identified carnivore’s scats genetically and then determined their diet using the classical approaches [27], [28], but this method has high costs associated. Even though field work has been done by experience personnel, with caution discriminating between wolf and domestic dog (*Canis lupus familiaris*) scats, it is always possible that a small number could belong to dogs, thus leading to an overestimation of the proportion of domestic animals in wolves’ diet. We tried to control this by doing genetic analysis on fresh collected scats and 4% samples ended up being excluded for belonging to domestic dog. Nevertheless, we believe that misidentification of wolf scats was minimized as dogs are mostly concentrated around villages. Furthermore, morphology, size, color, smell, contents and spatial position were, in combination, diagnostic attributes of wolf scats.

### Laboratory analysis

Prior to the laboratory analysis of the scats, we collected reference hair samples from all the livestock species present in the study area and also from domestic dogs, which are occasionally preyed by Iberian wolves [4]. We built reference slides of hair samples from domestic animals in the study area. In the laboratory, analyses were divided into two steps: scat washing and hair identification using standard procedures [29]. In the first stage, scats were washed with water and examined macroscopically, allowing the estimation of the relative percentage of animal matter (hair, bones, feathers, others), vegetable parts (grasses, seeds, others), mineral substances and garbage. In the next stage, hairs were identified using three techniques: cuticular slides, medullar slides and cross-section slides [29]. Then, the preparations were microscopically observed and compared with reference material (Valente et al. unpublished data) in order to identify the species. Hair identification was the only technique used to determine prey species; other animals were not classified to finer taxonomic levels because their remains in scats were often too fragmented. Scats that were highly degraded or had too few identifiable prey remains were not used in the analysis.

### Wolf diet composition analysis

The composition of food was expressed as frequency of occurrence (FO); calculated as the ratio between the number of scats in which a particular food item was found in the scats divided by the total number of scats examined. FO is the most commonly used method of diet analysis, however it may overestimate the frequency of small preys as smaller prey species have more hair and other indigestible matter per unit body mass, which produces more scats per unit prey mass consumed [30]. To convert the FO into biomass of prey consumed we used the regression method of Floyd et al. 1978 [30], as refined by Weaver biomass model [31]:

y=0.439+0.008*x

In this model, *y* is the prey mass consumed per scat and *x* is the average weight of an individual of a given prey type. By multiplying each *y* by the actual number of scats containing each prey type, we estimated the mass of each of the prey type in the scats and derived proportional representation. Body masses of the following prey species were estimated based on the literature: goat (30 kg), sheep (40 kg), cow (400 kg), lagomorphs (2 kg) and wild boar (70 kg) [32], [33].

The breadth of the food niche was calculated as described by Levins’ (1968) [34] according to formula:

$$B=\frac{1}{{\sum_{}^{}p}_{j}^{2}}$$

Where B = Levins' measure of niche breadth

p_j_ = is the proportion of usage of the *j* ^th^ prey item

To standardize this measure of niche breadth on a scale of 0 to 1, we calculated Levins’ measure of standardized niche breadth, according to the following formula [35]:

$$B_{A}=\frac{B-1}{n-1}$$

where B_A_ = Levin’s standardized Food Niche Breadth

B = Levin’s Food Niche Breadth

n = number of prey items found in the diet.

The Levin's standardized food niche breadth ranges from 0 (strong specialization in one group of prey—specialist predator) to 1 (opportunistic preying on all groups of prey).

To determine the degree of dietary overlap between the three packs we used Pianka’s (1973) dietary niche overlap [36] according to the formula:

$${\hat{\text{O}}}_{\text{jk}}=\frac{\sum_{i}^{n}{\hat{p}}_{ij}{\hat{p}}_{ik}}{\sqrt{\sum_{i}^{n}{\hat{p}}_{ij}{}^{2}\sum_{i}^{n}{\hat{p}}_{ik}{}^{2}}}$$

where:

O_jk_ = Pianka’s measure of niche overlap between species j and species k

p_ij_ = Proportion resource i is of the total resources used by species j

p_ik_ = Proportion resource i is of the total resources used by species k

n = Total number of resources states

This measure of overlap ranges from 0 (no resources used in common) to 1 (complete overlap).

Finally, to assess wolves’ selection of particular domestic species, we calculated the Ivlev’s electivity index (D) (modified by [37]) using the formula:

$$D=\frac{\left(r_{i}-p_{i}\right)}{\left(r_{i}+p_{i}-2r_{i}p_{i}\right)}$$

where: r = proportion of a given prey species in wolf diet;

p = proportion in the free living population.

The index ranges between −1 (total avoidance of a species) through 0 (no selection or selection proportional to occurrence) to +1 (maximum positive selection).

Structure of livestock communities in the areas occupied by the three packs was estimated using official data from the National Institute of Statistic [23]. The Ivlev’s electivity index could not be estimated for wild boar because there was no data available from the official game inventories for our study area.

## Results

**Fig 2:**
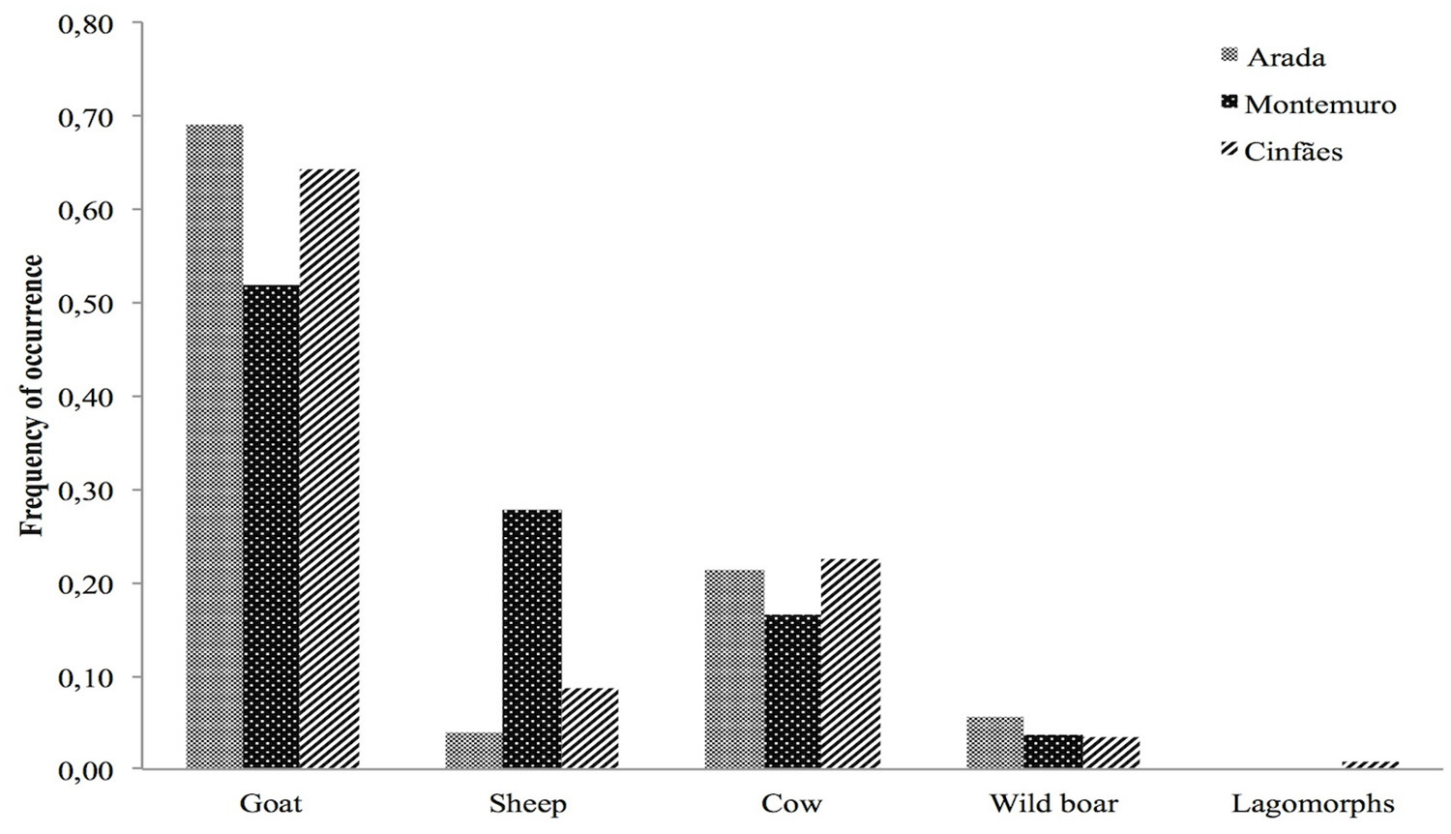
Composition of Iberian wolf diet in terms of frequency of occurrence, based on scat analysis of three Iberian wolf packs in central Portugal, between 2011 and 2014.

**Fig 3:**
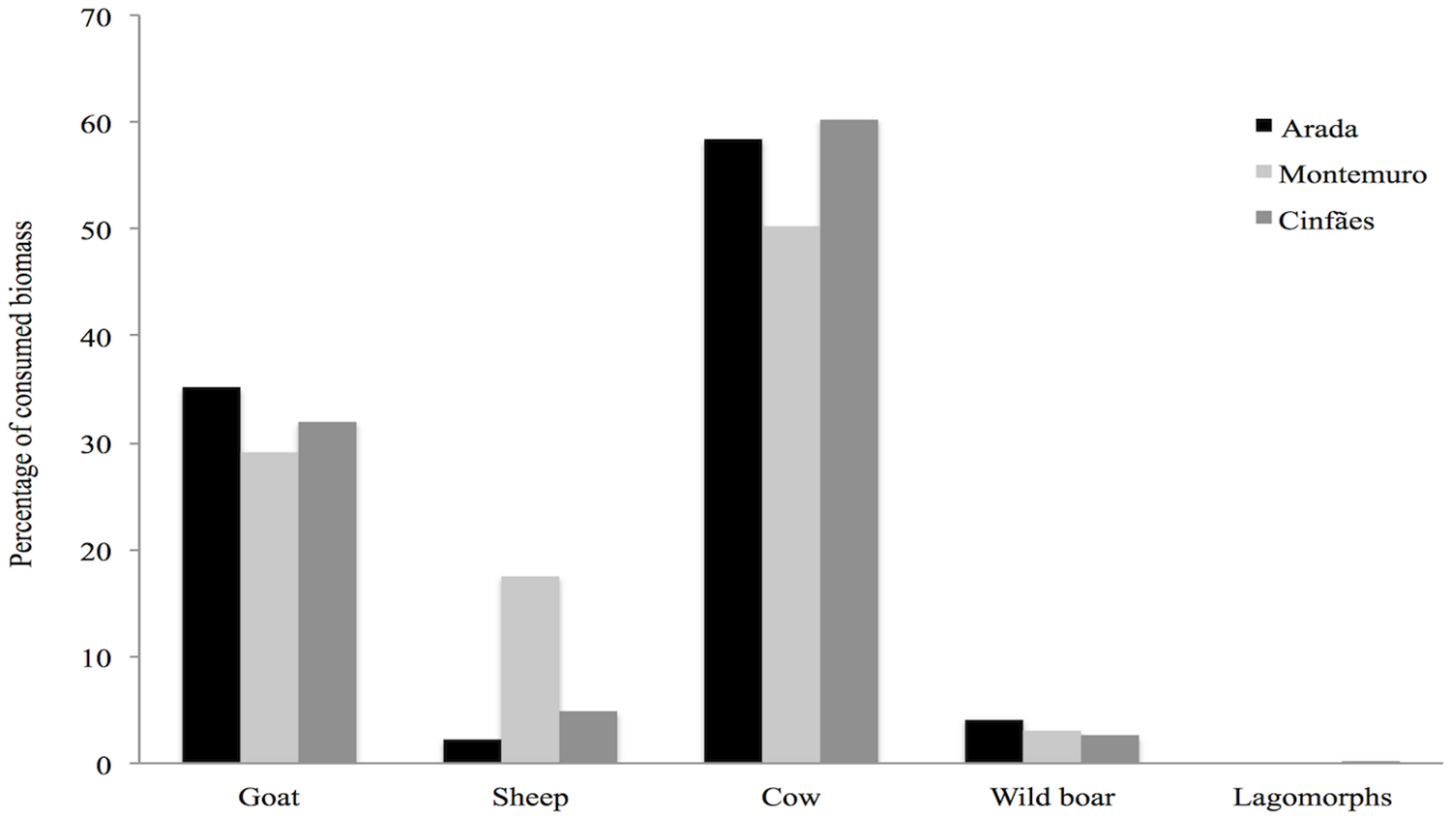
Prey species identified in the diet of Iberian wolf in central Portugal, in terms of biomass consumed, from analysis of 295 scats, collected from a human-dominated landscape between 2011 and 2014.

From October 2011 to April 2014, a total of 295 presumed wolf scats were collected in our study area: Arada pack n = 126, Montemuro pack n = 54 and Cinfães pack n = 115. The diet composition of the three packs was not diverse; a total of five different prey items (goat, sheep, cow, wild boar and lagomorphs) were identified in the diet. The diet of the three packs is given in Fig 2, showing the composition based on the frequency of occurrence of prey remains in scats. On the basis of the frequency of occurrence in scats, the domestic goat was observed to be the most consumed prey (69.05% in Arada pack, 51.85% in Montemuro pack and 64.35% in Cinfães pack), followed by cow (21.43% in Arada pack, 16.67% in Montemuro pack and 22.61% in Cinfães pack) and sheep (3.96% in Arada pack, 27.78% in Montemuro pack and 8.71% in Cinfães pack) (Fig 2). Regarding wild prey, wild boar was the most consumed (5.56% in Arada pack, 3.70% in Montemuro pack and 3.48% in Cinfães pack), followed by lagomorphs (1% only in Cinfães pack) (Fig 2). However, when the percentage of consumed biomass is considered, cow ranked the first of the consumed prey (56.34%), then goat (32.12%) and then sheep (8.19%) (Fig 3). Overall, domestic ungulates comprised the dominant part of the diet (94.45% in Arada pack, 96,3% in Montemuro pack and 95.67% in Cinfães pack) and wild species only a small part (5.56% in Arada pack, 3.70% in Montemuro pack and 4.34% in Cinfães pack).

Niche breadth index, estimated by the standardized Levin’s index, was narrow for all the three packs, indicating a specialization of wolves in one group of prey—livestock. Niche breadth index was greater for Montemuro pack (B = 0.56), and similar for Arada (B = 0.3) and Cinfães pack (B = 0.28).

The dietary overlap between the three packs, measured by the Pianka Index, was very high: 0.915 between Arada and Montemuro packs, 0.996 between Arada and Cinfães pack and 0,941 between Montemuro and Cinfães pack; the average was 0.931.

**Fig 4:**
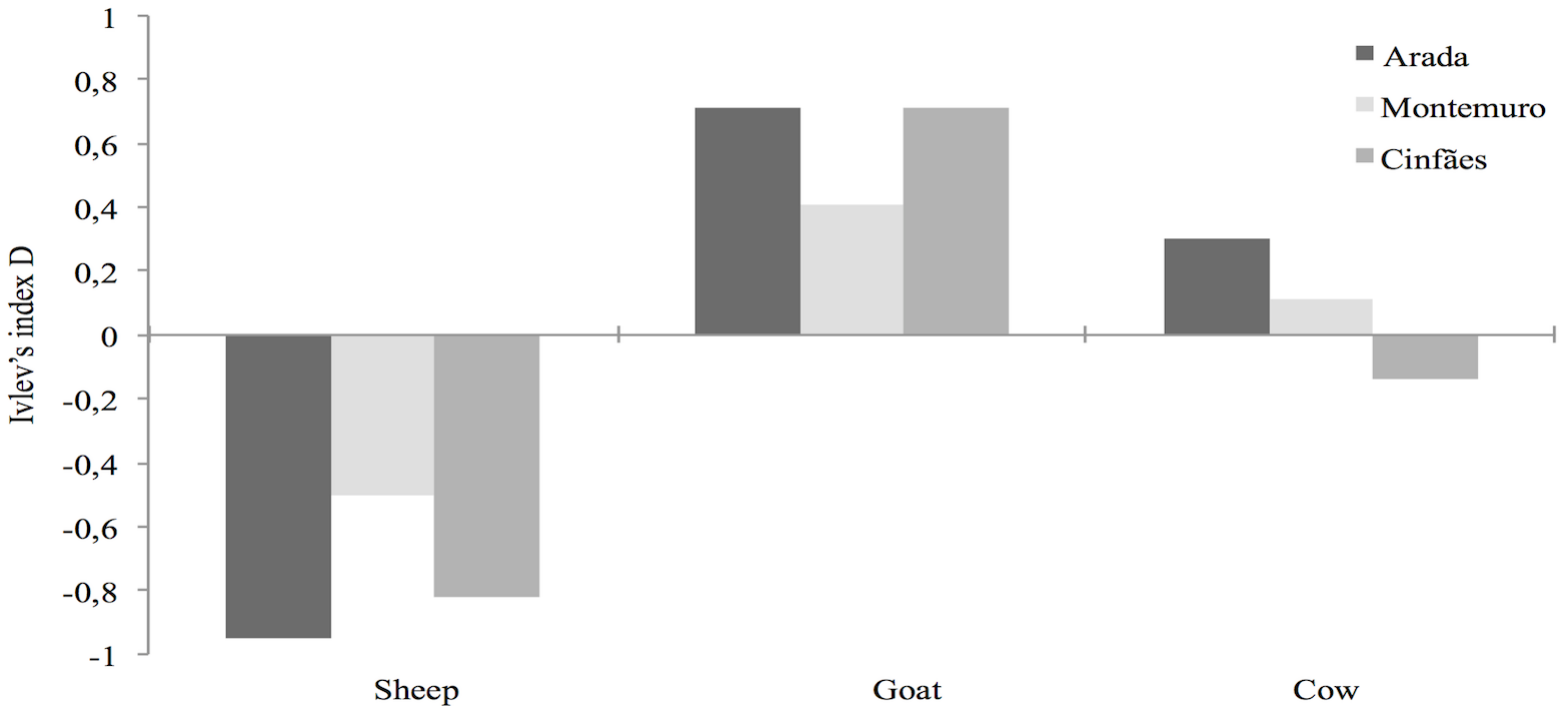
Prey selectivity (Ivlev’s index D) for sheep *Ovis aries*, goat *Capra hircus* and cow *Bos taurus*, based on scat analysis (n = 295) of three Iberian wolf packs (Arada, Montemuro and Cinfães), in central Portugal, between 2011 and 2014. The index is based on the frequency of prey species in scats relative to the availability of these species in the area. Species with positive index values are selected while those with negative numbers are avoided.

Ivlev’s electivity index showed that goat were positively selected in all packs (Arada: D = 0.71; Montemuro: D = 0.41; Cinfães: D = 0.71), being consumed upon a greater extent than expected by its availability, which could be attributable to preference or greater accessibility, cow was positively selected in the Arada pack (E = 0.3) and Montemuro pack (E = 0.11) but negatively selected in Cinfães pack (E = -0.14) and sheep was negatively selected in all packs (Arada: E = −0.95; Montemuro: E = −0.5; Cinfães: E = −0.82), being consumed less often than it might be expected from their availability in the study area (Fig 4).

## Discussion

Our study confirmed domestic ungulates as wolves’ primary prey in central Portugal. Such preference had already been shown in other areas of their southern European geographical distribution [14], [17], [18]. However, hitherto, no other study has reported such a extreme preference/dependence on domestic animals, with livestock constituting more than 90% of this species’ diet. Such fact is the reason for the rooted human-predator conflict in our study area, which can jeopardize the Iberian wolf conservation, ultimately resulting in some local extinctions. Nevertheless, [14] and [38] had already recorded high levels of livestock depredation by wolf in Portugal and central Iran, respectively. One of the reasons that might explain why livestock contributed to most of the wolf diet is the low diversity (and density) of wild ungulates. The main ungulate species in the study area is wild boar and that is reflected on our results. Contrarily, in central and eastern Europe wolves predominantly prey on wild ungulates [10], [12], [39], [40], [41]. When this occurs, there is generally a positive selection towards red deer [9] and roe deer [10]. Some studies showed a positive selection for wild boar in places where red deer densities were low [41], [42]. In a study in the Apennine mountains (Italy) wild boar constituted 62% of the consumed biomass [41]. In our study area, the lack of a diverse community of wild ungulates may lead wolves to feed on the only available wild ungulate. When there is a high abundance, richness and diversity of wild ungulate species, predation of livestock species is apparently low [13]. The other factor explaining high levels of livestock depredation is related to the husbandry practices. Livestock generally roams freely in the mountains in large numbers, representing a predictable and easy to kill prey that lack most anti-predator tactics. Some flocks in the study area roam alone, without any shepherd or guarding dogs. Studies have shown that improving efficiency of husbandry practices (confining sheep in fenced fields, as an example) dramatically reduces depredation losses by carnivore’s predation [43]. Wolves are opportunistic predators and are well adapted to prey overabundant livestock having done that for centuries in the humanized landscape of southern Europe.

In all the studied packs, based on the frequency of occurrence, wolves diet was based primarily on goats and this prey item was positively selected in all packs. This may be related to the presence of large flocks of goats (one village in our study area has more than 1.000 goats in just one flock) and their tendency to spread all over the mountain making them accessible prey. Interestingly, sheep were less consumed than expected. Wolves’ preference toward goats over sheep has been previously documented in Portugal [14] and Greece [18]. In central Italy instead, sheep was the preferred livestock prey, but in that case, goats’ availability was low [44]. As proposed by [45], prey selectivity depends on the degree of habitat overlap, encounter rates between predator and prey, vulnerability and predictability of prey occurrence. Maybe wolves have higher encounter rates and better attack opportunities with goat flocks as they are highly dispersed. This might be the case in our study area: goat flocks extent over larger areas and feed on remote and steep slopes, favoring wolves predation, on the contrary to sheep which roam together, with shepherds or guarding dogs and on more accessible areas. Additionally, wolves also prefer more inaccessible areas, representing less disturbed places, thereby overlapping its range with goats range as predation on goats must be higher in the most rough and remote parts of our study areas. Our results show that even though livestock constitute most part of wolves diet, different livestock species represent different predation opportunities for wolves. Comparing the three domestic species present in the study area, cows were the less consumed and even avoided in the Cinfães pack. Cows are the most valuable livestock species for rural communities, and shepherds might prevent cow predation by keeping cows closer to villages or by herding them. Additionally, cows are large preys and thus more dangerous or harder to kill and wolves might avoid the danger of getting injured if they have other predation opportunities. Transects were randomly distributed along the study area so we believe that our results are representative of the diet of the three studied packs and are unlikely to have bias. It is important to mention that scat analysis only reveals what wolves ate and that may not necessarily correspond to what they killed, as scavenging events on dead livestock can occur. In fact, one drawback of scats analysis is that it does not provide specific details about wolf predation and may overrepresent scavenged prey. However our results indicate a strong preference on livestock, and in our study area dead livestock are not generally left on the mountains for scavenging, so there are no reasons to believe that livestock presence in the wolf diet does not reflect wolf predation.

Our results showed that wolves in Portugal depend largely on livestock. Although wolves take advantage of the availability of food due to human proximity, they pay a high price by suffering a significant non-natural mortality. Moreover, in the last decades, there was a dramatic rural exodus, with many villages being abandoned in many regions of Portugal with a consequent decrease of livestock densities. With the main food source for wolves decreasing, and no wild prey being available, wolves may disappear from these regions. The scarcity of wild prey and human-conflicts can ultimately result in some local extinction. Wolves living in such conditions are in a precarious balance. In this context, a reintroduction project of roe deer is beginning in the same areas of the studied packs [46]. In a medium to long-term this will once again allow the wolf a choice of natural prey, and, politically, it will show that wolf conservation is a dynamic process and not merely a passive protection defense. Further, several studies have suggested that the presence of several wild prey species appeared to be more effective than a single prey in reducing livestock predation [13]. Livestock depredation seems to decrease in areas with higher densities of wild prey [13], [47]. However, the restoration of native ungulates will still take time; meanwhile with livestock decreasing in some regions of Portugal and no large wild prey populations being available, wolves may disappear from these areas. So, the short-term solution should not only be focused on reintroducing wild prey but additional management measures are needed. Even tough some controversy exists in the effectiveness of schemes that provide compensation for livestock losses to wolves, those programs can be helpful as short-term measures to mitigate the conflict between humans and wolves. We believe that changing attitudes towards wolves is urgently needed and environmental education is an important tool to get wolves accepted. But research on more appropriate ways of reducing livestock attacks by wolves is needed. Therefore strategies for balancing wolf conservation with human concerns are crucial. Based on what was previously mentioned it is obvious that a single solution is not the answer and resolutions have to be case specific. It is also fundamental a new culture of cattle grazing, including higher investment in livestock guarding dogs as they play a vital role in preventing livestock predation [48]. Several studies have showed that guarding dogs appear to reduce losses [48], [49]. Wolves may avoid areas with livestock guarding dogs, as dogs may disrupt depredatory sequences by wolves, enforcing indirect or direct aggression. In Portugal, some native livestock guarding dog breeds have been delivered to shepherds in the north and central Portugal. So far, the results are promising (reduction of the attacks from 13 to 100%) (S. Ribeiro, personal communication). However such number does not take into account some confounding factors (*e*.*g*. density of predators, livestock vulnerability, guarding dogs variability and breeds, experience of shepherds, etc).

The conservation of Iberian wolf populations should represent a priority in Portugal, where this subspecies is endangered. Strategies for balancing wolf conservation with human concerns are crucial for successful restoration and subsequent management.
